# Classification of Animal Movement Behavior through Residence in Space and Time

**DOI:** 10.1371/journal.pone.0168513

**Published:** 2017-01-03

**Authors:** Leigh G. Torres, Rachael A. Orben, Irina Tolkova, David R. Thompson

**Affiliations:** 1 Department of Fisheries and Wildlife, Marine Mammal Institute, Oregon State University, Hatfield Marine Science Center, Newport, Oregon, United States of America; 2 Applied Math and Computer Science Departments, University of Washington, Seattle, Washington, United States of America; 3 National Institute of Water and Atmospheric Research Ltd., Hataitai, Wellington, New Zealand; University of Alberta, CANADA

## Abstract

Identification and classification of behavior states in animal movement data can be complex, temporally biased, time-intensive, scale-dependent, and unstandardized across studies and taxa. Large movement datasets are increasingly common and there is a need for efficient methods of data exploration that adjust to the individual variability of each track. We present the Residence in Space and Time (RST) method to classify behavior patterns in movement data based on the concept that behavior states can be partitioned by the amount of space and time occupied in an area of constant scale. Using normalized values of Residence Time and Residence Distance within a constant search radius, RST is able to differentiate behavior patterns that are time-intensive (e.g., rest), time & distance-intensive (e.g., area restricted search), and transit (short time and distance). We use grey-headed albatross (*Thalassarche chrysostoma*) GPS tracks to demonstrate RST’s ability to classify behavior patterns and adjust to the inherent scale and individuality of each track. Next, we evaluate RST’s ability to discriminate between behavior states relative to other classical movement metrics. We then temporally sub-sample albatross track data to illustrate RST’s response to less resolved data. Finally, we evaluate RST’s performance using datasets from four taxa with diverse ecology, functional scales, ecosystems, and data-types. We conclude that RST is a robust, rapid, and flexible method for detailed exploratory analysis and meta-analyses of behavioral states in animal movement data based on its ability to integrate distance and time measurements into one descriptive metric of behavior groupings. Given the increasing amount of animal movement data collected, it is timely and useful to implement a consistent metric of behavior classification to enable efficient and comparative analyses. Overall, the application of RST to objectively explore and compare behavior patterns in movement data can enhance our fine- and broad- scale understanding of animal movement ecology.

## Introduction

**Fig 1:**
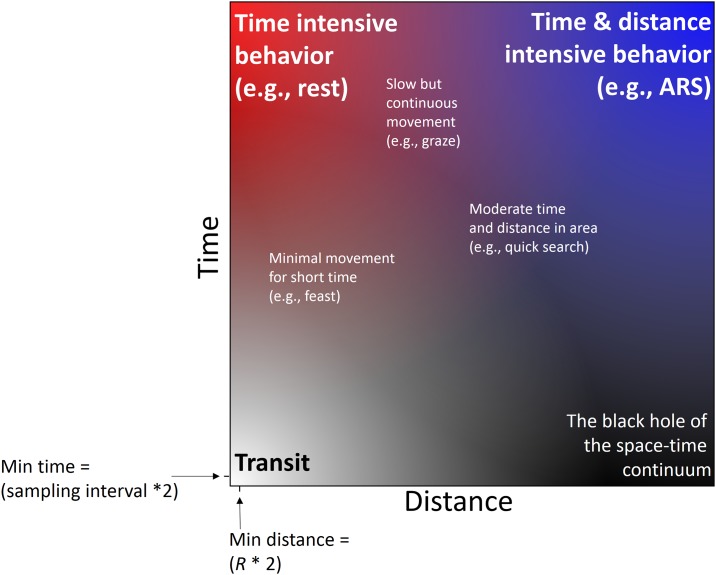
Conceptual schematic of behavior groupings captured in movement data based on the relationships between the amount of space (distance) and time occupied in an area of constant scale. Three polar behavior states across this continuum are represented in the corners: Transit (low time, low distance in an area), time intensive behaviors such as rest (high time, low distance), and time and distance intensive behaviors (high time, large distance) such as area restricted search (ARS). Three other possible behavior states are denoted within the continuum of this schematic. When applying RST, the origin will be double the sampling interval (y-axis) and double the *R* applied (x-axis), which are the minimal scales at which behaviors can be described.

Time and space are fundamental to animal ecology, as these factors limit and scale behavior patterns. Animal-borne location tags are prolifically used to capture animal movement in both of these dimensions, yet behavioral analyses of these data have primarily focused on the assessment of temporal patterns across space (i.e., first passage time [1]; residence time [2]; time-in-grid [3]). While informative, the omission of analogous cumulative spatial metrics limits the ability of these methods to discriminate between time intensive behaviors such as rest and area restricted search (ARS; [1, 4]), which can have variable distance values, but similar time values. Additionally, commonly applied imputs to describe behavior states such as step-length and turning angle are often constranind to the scale of the sampling interval rather than a scale selected based on the movement or perception of the animal (behavioral change point analysis [5]; hidden Markov models [6]). Therefore, classification of behaviors can be enhanced by describing both spatial and temporal occupancy patterns, while also considering both the temporal and spatial scale of the analysis. To illustrate this, consider an area of constant scale (e.g., 1 x 1 km), within which animal behaviors differentiate based on the relationships between the total distance traversed and the amount of time spent in the area of constant scale (Fig 1). The axes of this schematic scale from low to high distance (x-axis) or time (y-axis) so that when an animal’s spatial and temporal occupancy patterns are related, behavioral groupings emerge. The corners of this schematic represent the polar, dichotomous behavior states of (1) transit, near the origin, where the animal incurs low time and low distance in the area, (2) time intensive behaviors such as rest, in the upper left, where the animal incurs high time in the area but covers little distance, and (3) time & distance intensive behaviors in the upper right where the animal incurs high time and distance covered within the area, representing behaviors such as ARS that are influenced by any combination of reduced speed, increased turning, and increased time spent in the area. Given the inability to move large distances in short time periods (teleportation), it is impossible to fall within the ‘black hole’ of our schematic in the bottom right. Within the boundaries of these three dichotomous behavior groupings, multiple other behavior states can be identified and grouped also based on the comparative amount of time and space within the area such as graze, feast, and quick search.

Examination of behavioral subsets of movement data allows focused and comparative studies. Thus, behavior classification is often an early and critical component to movement data analysis that guides further analysis pathways. While behavioral interpretation is often intuitive upon visual assessment of each track, the classification of behavior states can be difficult to automate and objectively quantify. Many quantitative methods to classify behavior states are in use but, in addition to being biased toward temporal metrics, these are often statistically complex (e.g., Bayesian state-space models [7]; biased random bridges [8]; tortuosity entropy [9]) or require advanced programming skills and ample time to run the models, especially for first-time users (e.g., hidden Markov models [3]; wavelet analysis [10]). Therefore, there is a need for a simple and quick method to explore, segment, and behaviorally annotate movement data with limited supervision [11]. Additionally, these methods may lack transferability between taxa or studies, or be difficult to successfully apply to large and varied datasets with high individual variability [12]. These challenges are becoming increasingly salient with the increasing number and size of animal movement datasets [13] due to miniaturization, and increased resolution, memory capacity, and battery life. Over 3,500 animal movement studies containing over 260 million locations have been contributed to movebank.org, seabirdtracking.org, and OBIS-SEAMAP (tabulated on 31 March 2016). The growth of biotelemetry offers immense opportunities for discovery, yet ‘methodological ambiguity’ for data exploration leads to confusion and inconsistency [14] and movement ecologists may struggle to balance the analytical demands of Big Data [15] with the individuality of each track. In this study, we offer an efficient, objective and broadly applicable method to explore and identify behavior patterns at multiple scales in movement data.

Building off the concept of residence time [2], we first develop a metric of residence distance. These two metrics quantify cumulative area occupancy in time and distance respectively, and when related to each other, behavioral groups can be discerned (Fig 1). The method identifies three fundamental movement states: transit, time intensive movement, and time & distance intensive movement. These states are identified on a continuous scale that can be applied in further *post hoc* analyses. Initially, we develop and test our Residence in Space and Time (RST) method using a highly resolved grey-headed albatross (*Thalassarche chrysostoma*) GPS track. We discuss the impact of scale on RST behavior classifications and present methods to evaluate scale choice. Next, we demonstrate the ability of RST to discriminate between three discrete behavior states of an albatross (rest, ARS and transit) relative to other classical movement metrics. The RST method is then applied to 24 albatross tracks to assess the method’s ability to describe population-level behavior grouping while assessing individual variation. Next, we explore RST’s ability to accurately describe behavior states in movement data from less temporally resolved and temporally intermittent datasets (mimicking Argos/PTT tracks). Finally, we apply the RST method to animal movement datasets from diverse taxa and ecosystems to evaluate performance and versatility. This exploration demonstrates that RST is flexible and robust for application to multiple taxa and movement data types, which allows an efficient initial data exploration method to inform subsequent hypothesis testing, data partitioning, and appropriate analyses.

## Materials and Methods

### Ethics statement

All handling of albatross was conducted under permit issued by the New Zealand Department of Conservation and was approved by the NIWA animal ethics committee. All effort was made to minimize handling time and any suffering to animals.

### RST development and dataset

During October and November 2013, grey-headed albatrosses breeding at Campbell Island in the New Zealand sub-Antarctic were tagged with igotU GPS archival tags (GT-600; http://www.i-gotu.com/), set to record a position and time every five minutes. We recorded incubation foraging trips of adult albatross (n = 24) after securing the GPS tag to back feathers using Tesa^®^ tape. To focus on at-sea behaviors we removed all points within 5 km of the colony [16]. We completed all analysis in R [17] and implemented in C, with adapted code from Chirico [18] and Kahle and Wickham [19].

We then calculated residence distance (RD) and residence time (RT) for all points along the track. A circle of radius *R* is constructed around every point and the distance traveled (RD; sum of path lengths within the circle) and time spent (RT; sum of time between locations within the circle) between consecutive points within the circle is calculated. Unlike Barraquand and Benhamou (2) Residence Time method, our calculations of RT and RD do not include the ‘tails’, which are the path segments between the first or last point in the circle and the perimeter. With our approach, all points alone within the circle are assigned a value of zero for both RT and RD. If the path trajectory exits and reenters the circle with no more than a threshold distance value (*Th*) traveled outside, the stretches of track outside the circle are also included in the RD and RT values. We include the option to set a threshold distance in the RST method for consistency with the original Residence Time method [2], yet within the RST method its functionality for behavior classification is limited. Therefore, in the following examples we set *Th* equal to zero.

To test the hypothesis that variation between RT and RD is related to movement behavior, we calculated the residuals (difference in value) between these metrics for each point. First, RD and RT values were normalized by dividing by the maximum respective value within each track so that distance and time values were unit-less and therefore comparable, and so all values consistently ranged between 0 and 1. Then residuals for each location were calculated by subtracting RT from RD. To complete these steps the following formula was applied:

Residuals = ((RD) / (max. RD of the track)) − ((RT) / (max. RT of the track)) (1)

We used the difference between RD and RT to describe behavior patterns, rather than proportion, sum, or other complex comparison, because this approach (1) results in a consistent range of residuals between -1 and 1 that is comparable between individuals and datasets, and (2) allows for a relatively limited chance that the same value will result from different combinations of RD and RT (S1 Appendix). Speed also describes the relationship between distance and time, but is not directly suitable for behavior classification because speeds at large and small scales can be equivalent and therefore difficult to relate to behavior states.

The scale-dependence of RST relates to *R*, and both RD and RT assign zero to locations that are > *R* away from other points. The appropriate *R* value depends on the temporal sampling interval and animal behavior patterns captured by the data. We offer two approaches to the selection of *R* based on animal transit speed. Transiting is a fundamental and shared behavior between animals, which is constrained by physiology, morphology and environment. Therefore, with RST, transit points separate positive (time & distance intensive) and negative (time intensive) residuals, so that the classification of transit points influences the behavior types described. One approach to *R* selection is derived by the following formula:

R=(mean transit speed * sampling interval)/2 (2)

which assumes that the average distance between transit points should be approximately equal to the average transit speed multiplied by the sampling rate, and divided by two to uncouple two consecutive points. This approach assumes *a priori* knowledge of transit speed. Alternatively we apply a diagnostic tool to calculate the percent of points with positive, negative and zero residual values at multiple (user defined) scales to assess the impact of *R* selection. We apply Formula 2 again and determine the numerator as the scale where the number of transit points approaches zero (where all points have at least one other point inside its circle). Extremely fast movements or large data gaps prevent this value from actually reaching zero, so we use <5% transit points as the cutoff. A benefit to this approach is automated dynamic scaling for each track.

### Example application of RST to one albatross track

Grey-headed albatrosses have three dominant and discrete behavior states at-sea: transit, ARS foraging, and rest; which are linked to strong diurnal patterns of limited activity during darkness [20]. We illustrate the behavioral classification capability of RST using one albatross GPS track (Bird 23059) by assessing the relationship between RD and RT, and the variation in residual values relative to day and night. A static *R* of 1.935 was applied based on Formula 2, using a mean transit speed of 45 km/hr [21] and mean time interval between locations of 5.16 ± 1.0 min. The dynamic scaling approach was also applied to this albatross track for comparison of radii values.

### Comparison of metrics in three behavior states

RST’s ability to discriminate between three discrete behavior states along this grey headed albatross track (Bird 23059) was directly compared to other classical movement metrics of speed, path straightness (straight-line distance between points / cumulative path lengths between points), and residence time and residence distance using the Barraquand and Benhamou [2] approach that includes the ‘tails’. Three experienced seabird ecologists very familiar with albatross movement data (L.G.T., R.A.O. and D.R.T.) manually and independently classified each GPS location into rest, transit or ARS behavior states. Without direct observation it is near impossible to know the true behavior state of a tracked animal. Therefore, we assumed the points with matching behavior state assignment between the three classifiers to be ‘true’, and compared frequency histograms of the movement metrics speed, path straightness, residence time, residence distance, and RST in the three behavior states rest, transit, and ARS. All metrics were calculated using an *R* = 1.935.

### From individual to population

To evaluate RST’s ability to classify behavior states within movement data from a sampled population, we applied the method to all albatross incubation trips (n = 24). We analyzed the albatross tracks with *Th* = 0 and (1) a constant *R* based on a transit speed of 45 km/hr and a mean GPS fix interval = 5.63 ± 0.59 min, and (2) using the dynamic scaling method for each track. As before, we assessed behavior classification based on residual variation relative to daylight. We also timed this analysis to demonstrate the method’s speed.

### Impact of temporal resolution on RST

To evaluate RST’s ability to classify behaviors using less temporally resolved data, we completed two subsampling exercises. First, we subsampled all albatross tracks at across a range of increasing temporal intervals (10, 20, 30, 60, 120, 180 min) and applied the dynamic scaling method to choose an appropriate *R* for each sampling interval and individual combination (S2 Appendix). Secondly, we stochastically subsampled the 60 min subsample of a single albatross track (Bird 23059) 100 times to randomly select 1/3 of the locations. These subsampled tracks mimic the erratic sampling of commonly used satellite telemetry. For each subsampled track, we calculated the percent of locations matching the residual state (positive, negative, or zero) of the original 5-min sampling interval track to assess the variance of behavior classification relative to temporal resolution of the tracking data.

### Application of RST to diverse taxa

To evaluate and expand the application of the RST method, we used movement datasets from four taxa with diverse life-history patterns (predator, prey, grazer, migrator), with variable home range scales, from terrestrial and marine ecosystems, and of different data types. Three datasets were freely downloaded from the Movebank Data Repository (https://www.movebank.org/), which has proven to be a powerful resource for our exercise: (1) a 2-month GPS track of a medium-sized carnivore, the fisher (*Martes pennanti*), tagged in New York, USA, in March. 2011, with dynamic sampling using tri-axial accelerometer data (2-min sampling when moving; 1-hr sampling when resting (tag M4 [22, 23]); (2) a 2-month GPS track of an African buffalo (*Syncerus caffer*) collected in Kruger National Park, South Africa, from 10 October to 7 December 2005, with 1-hr sampling interval (tag 1764827 [24]); (3) a 5-year GPS track of a Galapagos tortoise (*Chelonoidis vandenburghi*), tracked on Isabela Island, Galapagos, beginning in October. 2010, with 1-hr sampling intervals and a duty cycle shutdown period from 0100 to 1100 GMT when the animal is generally stationary (tag 1388 [25]). Additionally, we analyzed a satellite telemetry track of a blue whale (*Balaenoptera musculus*) tagged off Southern California, USA, with movement data from September 2007 to February 2008 (tag 23043 [26, 27]). We analyzed these four datasets using the RST method and a dynamic scaling approach (*Th* = 0), as we assumed no *a priori* knowledge of animal transit speed.

## Results

### Application of RST to one albatross track

**Fig 2:**
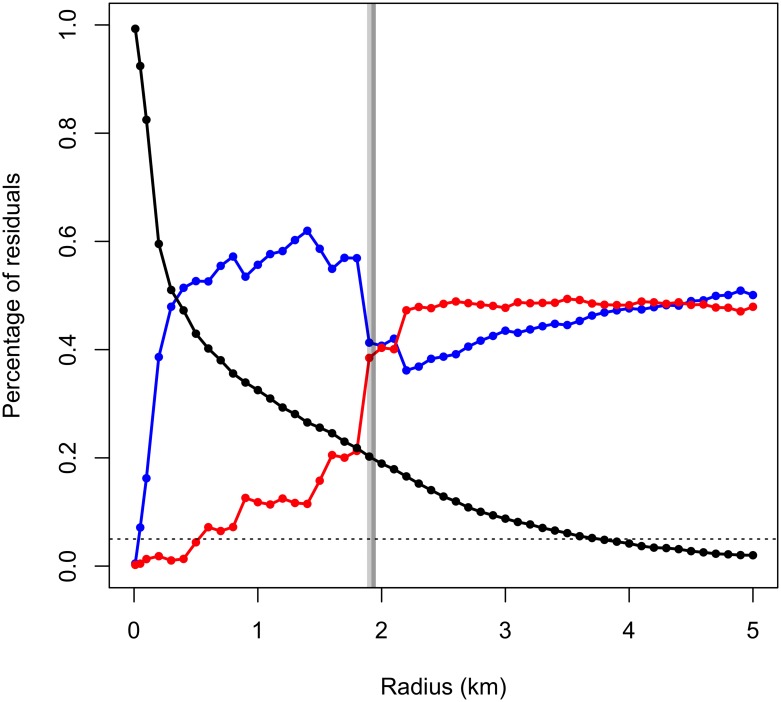
Scale plot of grey-headed albatross GPS track illustrating how radius size influences the proportion of positive (blue), negative (red) and zero (black) residuals. Dark gray bar = fixed radius (*R* = 1.935). Light gray bar = dynamically scaled radius (*R* = 1.9). Dashed line indicates 5% transit points.

A very similar *R* value of 1.9 was selected by the dynamic scaling approach when applied to the albatross track compared to the static *R* value calculated through Formula 2 (*R* = 1.935). The resulting scale plot (Fig 2) illustrates that as *R* increases the number of transit points decreases while positive and negative residuals increase.

**Fig 3:**
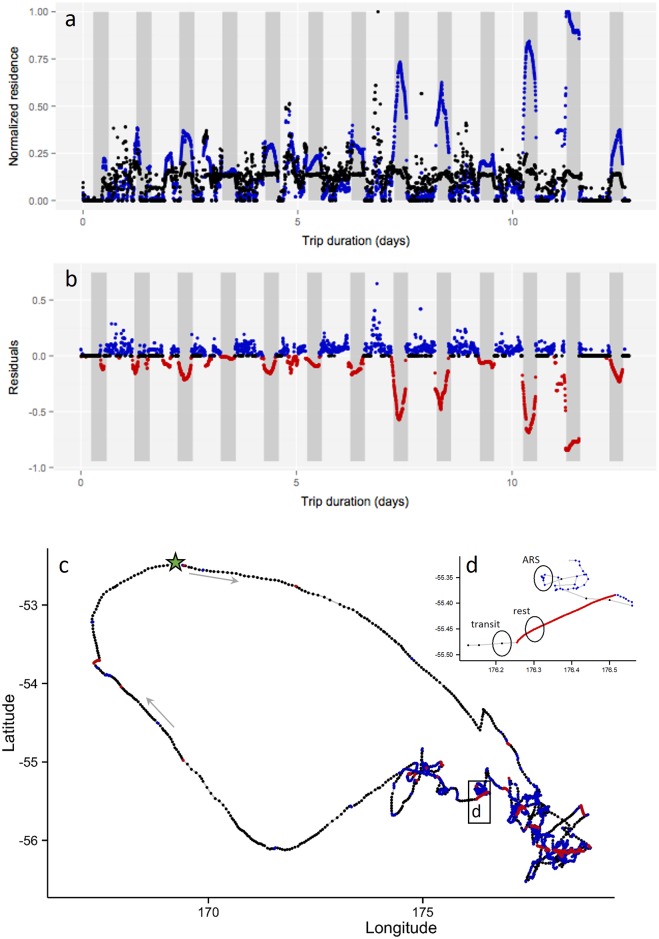
RST analysis of example grey-headed albatross GPS track. Day and night (shaded) periods compared to (a) normalized residence distance (black) relative to normalized residence time (blue), and (b) residuals of normalized residence distance minus normalized residence time (positive = blue, negative = red; zero = black). (c) GPS track color coded by residuals (black = transit, red = rest, blue = area restricted search). The three movement states identified by RST are illustrated and (d) enlarges a region of the track to demonstrate the classification of three locations into these movement states within the applied radius size. Grey arrows indicate direction of travel. Green star is colony location at Campbell Island, New Zealand.

Overall, the response of RD and RT to albatross track geometry agree during daylight (Fig 3a). However, during nighttime, RT values are elevated compared to RD values that remain at a more average value compared to daytime variation. The inflation of RT illustrates the behavioral bias of a time metric toward resting behavior, which albatross are generally engaged in at night. In contrast, RD is immune to this response. Yet, behavioral separation of the movement data is evident when RD and RT are compared using the RST method (Fig 3b). Time intensive behaviors, representing rest periods in this case, are evident at night with RT > RD, equaling negative residuals. Positive or zero value residuals generally occur during daylight, when albatross are travelling or engaged in ARS. Correspondence between behavior and residual groups is visually evident (Fig 3c and 3d) with transit between foraging areas (black), clustered ARS (blue), and interspersed rest segments (red).

### Comparison of metrics in three behavior states

**Fig 4:**
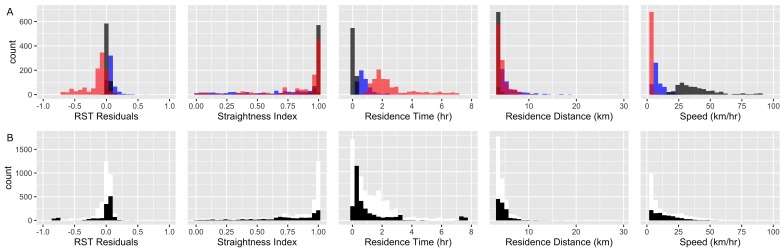
Frequency histograms of RST residuals relative to classical movement metrics (straightness index, residence time, residence distance, and speed,) for points along the grey-headed albatross track (Bird 23059). (a) Depicts only the ‘true’ behavior states of rest (red), transit (black), and area restricted search (blue) as agreed on by expert classifiers. Bars are colored based on RST classification with transparency so that overlap between distributions is illustrated. (b) Describes the distribution of all points along the track (white) and the ambiguous points where the classifiers did not agree on behavior state assignment (black).

Behavior states matched between the three expert classification efforts in 66% of locations (2336 of 3548 points; n = 708 transit; n = 1080 rest; n = 548 ARS), which were considered the ‘true’ behavior states. The variability in behavior state classification of the remaining 1212 ‘ambiguous’ points is likely due to (1) differences in the inferred scale of assessment by each classifier, (2) presence of points recorded during transitions between states, and (3) the inherent ambiguity of assigning points into one discrete behavior group that are simultaneously multiple behavior states (e.g., slightly sinuous travel, which can be interpreted as either transit and ARS). RST residuals aligned with our manual classification effort for 90% of the locations (2112 of 2336 points; Fig 4a). The majority of discrepancy occurred due to RSTs tendency to identify points as time & distance intensive movement (n = 143), while the classifiers labeled such points transit. Similarly, RST classified the majority of ambiguous points as time & distance intensive points (black bars in Fig 4b).

When compared to other time series metrics, RST residuals were able to discriminate between the three ‘true’ behavior states with little overlap. Residence time as calculated by Barraquand and Benhamou [2] also shows little overlap between ‘true’ behavioral states (Fig 4a). However, determining breakpoints of behavior states from the continuous range of residence time values is difficult (white bars Fig 4b). Furthermore high residence times does not equate to a distinct behavioral state (either rest or ARS). Speed is almost discrete between the three ‘true’ behavior states as color-coded by RST classification but, like residence time, is unable to independently group behavior states or classify the ambiguous points (Fig 4b). Path straightness and residence distance were both unable to distinguish between transit and time intensive behaviors because these points have relatively straight paths and low distance. Behavior classification based on RST benefits from its integration of multiple movement data measurements into one combined metric. Due to the calculation of metrics within an area, RST’s classification of each point depends on its neighboring points, which results in more stable behavior states compared to point-based approaches [11, 28] that produce more erratic behavior state switching between points.

### Population-level performance of RST

To evaluate the population-level performance of RST, all incubation albatross tracks (n = 93,481 locations) were analyzed. Using a fixed *R* = 2.11 km, behavioral classification of locations resulted in 28.0% transit (residual = 0), 48.8% ARS (residual > 0), and 23.2% rest (residual < 0). Using the dynamic scaling approach to determine *R* for each track (mean *R* = 2.55 ± 0.41 km), behavioral classification of locations resulted in 22.9% transit, 50.9% ARS, and 26.2% rest. Using a fixed radius and dynamic scaling, respectively, 74.4% and 76.5% of the negative residuals (rest) occurred at night, while 82.6% and 82.6% of positive residuals (ARS) occurred during the day. Similar *R* values, proportions of behavioral classifications, and diurnal behavioral assignment were determined by both methods of *R* selection, indicating that dynamic scaling can perform well if animal speed is unknown. Running the RST code to identify the dynamically scaled radii for each of 24 tracks using 44 radii options took 52 seconds (CPU time = 9 sec, Processor = 2.66 GHz Intel Core 2 Duo), and once the preferred radius for each track was identified, these 24 tracks took a mere 22 s (CPU time = 1.8 s) to compute.

### RST’s response to less temporally resolved data

**Fig 5:**
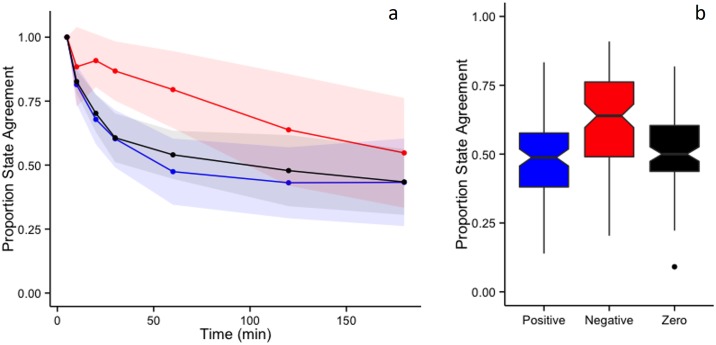
Behavioral state, based on positive, negative, or zero residuals, agreement plots relative to 5-min interval track for (a) population level temporal sub-sampling of all incubation albatross tracks (shaded areas represent SD), and (b) stochastic sampling of one albatross track (notch = median, whiskers represent 1.5 * inter-quartile range). Blue = area restricted search (positive residuals); red = rest (negative residuals); black = transit (zero residuals).

The RST behavior class (ARS: residuals > 0; rest: residuals < 0; transit: residuals = 0) agreement test between each location in the original 5-min interval track and the temporally subsampled tracks demonstrate the impact of behavior bout length on behavior class detection (Fig 5a). At longer time intervals, time intensive behaviors (rest) remain relatively well classified, but behaviors with shorter bout lengths (ARS and short transits) are increasingly misclassified as the sampling interval grows longer than the bout length (S2 Appendix). In this example, albatross ARS bouts appear to occur at temporal scales < 30 mins, and transit periods longer than 60 mins are consistently identified, which likely represent persistent travel to and from the colony (Fig 5a). The satellite telemetry simulation of stochastically sampled data reiterates this pattern: negative values (rest) remain well classified, while positive (ARS) and zero (transit) value residuals are misclassified more than half the time (Fig 5b; S2 Appendix). This exercise demonstrates that behavioral analysis of satellite telemetry data may indicate where animals spend greater time, but not necessarily where they conduct ARS. Speed filtered satellite telemetry data may reduce spatial error and provide more accuracy in behavior classification. Additionally, track interpolation would decrease the sampling interval, reducing *R* (Formula 1) and increasing the percent of transit points (Fig 2).

### RST analysis of diverse datasets

**Fig 6:**
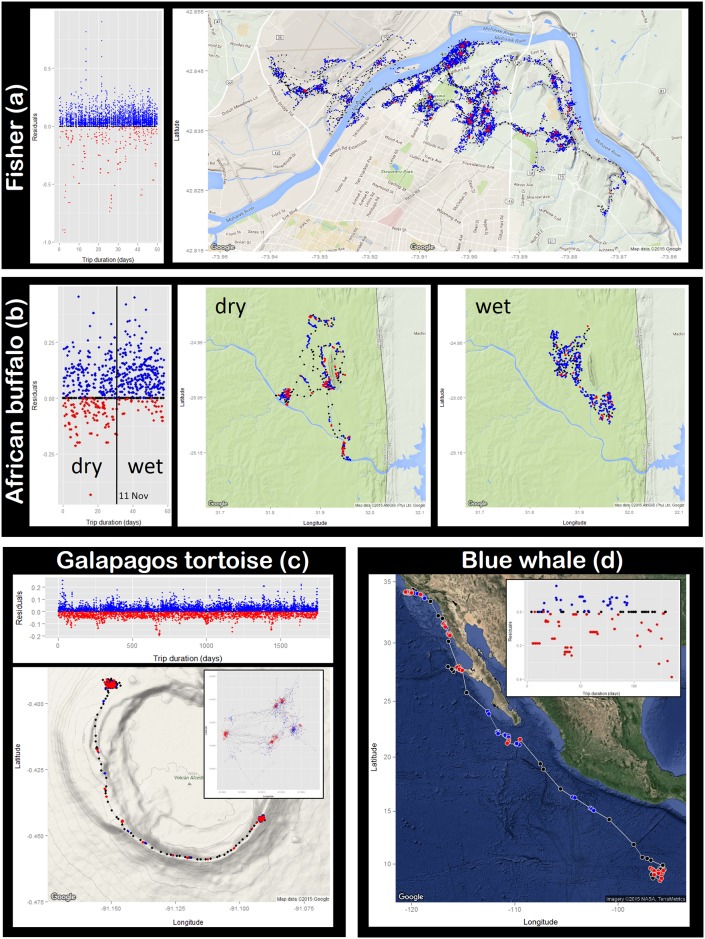
Application of RST to four diverse movement datasets. (a) 2-month GPS fisher track in an urban area of New York, USA, and residuals (tag M4 [22, 23]). (b) 2-month GPS African buffalo track and residuals split at 11 Nov 2005 to demonstrate behavior and distribution change with onset of wet season (tag 1764827 [24]). (c) Residuals from 5-year GPS Galapagos tortoise track, and spatial representation of track segment from 1 Aug 2011 to 30 Mar 2012; inset map shows fine-scale movements in southeastern area (tag 1388 [25]). (d) 5-month satellite telemetry blue whale track starting off southern California and ending near the Costa Rica Dome, and residuals (tag 23043 [26, 27]). Maps produced using R code by Kahle and Wickham [19].

Analysis of the high-resolution fisher track (*R* = 40 m) through an urban habitat, reflects discrete and clustered locations of periodic short-term resting places [29], with more dispersed searching/foraging locations interspersed with relatively linear transit segments (Fig 6a). RST classification of resting/stationary behavior states in this fisher track was not influenced by the less frequent GPS sampling caused by accelerometer-informed data loggers because RT is a cumulative measure of time spent within circle of radius *R* and therefore a resting fisher would accumulate the same RT value regardless of GPS sampling frequency.

RST analysis of the African buffalo track (*R* = 375 m) effectively describes transit locations between areas of increased RT or RD. Additionally, the RST analysis highlights a behavior shift around November 11 with the onset of the wet season (rains began in early Nov. 2005) to predominantly time & distance intensive behaviors (positive residuals; blue locations) and altered distribution patterns as the animal moves away from river beds and spends more time in the plains (Fig 6b), matching their known ecology [30]. Evaluation of the long-term tortoise track (*R* = 25 m) revealed oscillation of residual values and intensities relative to its location in NW and SE seasonal areas, indicating different movement strategies between habitats (Fig 6c). During one migration cycle depicted (Aug. 2011 –Mar. 2012), transit points are identified between the two areas, and fine-scale assessment of the SE area illustrates discrete areas of time intensive and time & distance intensive behaviors.

**Fig 7:**
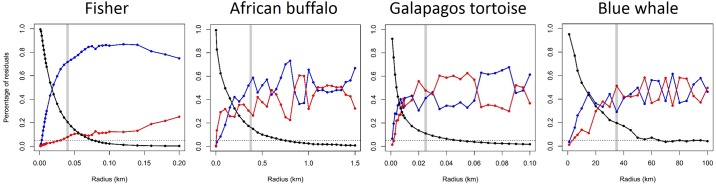
Scale plots derived using dynamic scaling choice of radius size (*R*) for Residence in Space and Time (RST) analysis of the fisher GPS track, African buffalo GPS track, Galapagos tortoise GPS track and blue whale satellite telemetry track. The comparison illustrates how *R* influences the proportion of positive (blue), negative (red) and zero (black) residuals. Dashed line indicates 5% transit points. Light gray line indicates the dynamically scaled *R* for each track: Fisher (*R* = 40 m), African buffalo (*R* = 375 m), Galapagos tortoise (*R* = 25 m), blue whale (*R* = 35 km).

RST analysis of the lower resolution blue whale track (*R* = 35 km) identifies alternating time intensive and time & distance intensive behaviors while foraging off Southern California and central Baja California, interspersed with transit periods (Fig 6d). The animal switches to mainly time & distance intensive behaviors off central Mexico, and then to transit behavior during migration toward the Costa Rica Dome where time intensive behavior is exhibited. At this scale of analysis, the shifts between time intensive and time & distance intensive behaviors may represent two different scales of area restricted searching by this whale. Considering the results of our satellite telemetry simulation, behaviors with bout lengths smaller than the temporal sampling may be misclassified, yet the results coincide with known blue whale ecology in this region [26]. Overall, the application of the RST method to these various movement datasets illustrates its flexibility and explanatory power. For each taxa, RST describes alternating behavior states that correspond to their known ecology, and comparatively reveals the fisher’s striking preference for distance intensive movement patterns (Fig 7).

## Discussion

Given the large and increasing amount of animal movement data collected, it is timely and useful to implement a consistent metric of behavior classification to enable efficient and comparative analyses. Indeed, movement ecology needs unifying paradigms to converge diverse studies and foster a mature scientific discipline [13]. The RST method offers a fast approach to the analysis of movement data that requires low computational power and time investment, while also allowing individualization by track using the dynamic scaling approach. Therefore, we advocate that RST is an effective and efficient method for initial exploration of movement data to inform hypothesis testing, data partitioning, and choice of modeling or statistical framework for subsequent analyses. Such close and detailed exploratory analysis of behavior state and scale before fitting complex movement models is critical as movements are often hierarchical and cyclical [14]. Furthermore, RST appears to be robust across taxa, ecosystems, and movement data types, and generates a consistent range of residual values that are comparable, making it an appropriate method of meta-analyses of movement data. RST is based on our conceptual schematic illustrating how the comparison of animal movement patterns through space and time are able to discriminate between behaviors states resolved in the data (Fig 1). RST is a composite of other movement analysis metrics (RT, RD, speed, and path straightness) that integrates these descriptions of movement patterns through both space and time to distinguish between multiple behavior states. RST allows behavior classification to move beyond the dichotomy of ‘travel’ and ‘resident’ (e.g., [3]), and is a one-step method of behavior classification, unlike many other methods that first necessitate metric calculation and then the application of a subsequent time-series or clustering algorithm to define breakpoints (e.g., [2, 5, 11, 28]). Our novel method is intuitive and simple to implement, offering a flexible framework to quickly and objectively characterize behavior states, point-by-point, in diverse movement data types.

The premise of all movement analyses is that animals change movement patterns relative to different behavior states. But ultimately it is the scale of analysis that determines the movement patterns described [31], and therefore the behaviors characterized. RST allows various scales (*R*) to be examined simultaneously, and we offer two approaches to help the researcher discern an appropriate scale. The first approach assumes *a priori* knowledge of the animal’s mean transit speed and would apply a constant scale across a single-taxa dataset. The dynamic scaling approach offers two benefits: (1) it allows for scale-dependent comparison of behavior states similar to Postlethwaite et al. [32] but with objective discrimination between behaviors, and (2) it adjusts *R* for each track, enabling flexibility of scale application that accounts for inherent individual movement patterns, such as speed and tag variability. Dynamic scaling prioritizes the classification of transit points, at all scales analyzed, and therefore performs best on tracks with some transit behavior.

Nonetheless, one scale is unlikely to be appropriate for long duration tracks with high sampling resolutions due to various behavior patterns layered in the data at multiple scales, and variable transit speeds during different life history stages. In such cases, tracks may be split by phase (e.g., migration, breeding, season) prior to final RST analysis, or multiple *R* can be applied to resolve behaviors at different scales. This is exemplified by our choice to limit RST analysis of albatross tracks to movement behavior at-sea. Alternatively, if we had included incubation periods (high RT, low RD), this would bias the RST values of at-sea resting behavior towards positive values, especially as resting at sea is not stationary. Ultimately, partitioning of tracks and scale choice is case-dependent and should be based on study questions, taxa, and environment. However, the primary determinant of minimum scale is data resolution. Only behaviors that occur at spatial and temporal scales larger than the sampling interval and spatial resolution of the movement data are recorded, and hence described. This effect is emphasized by our subsampling analysis. With less resolved data, behaviors with long bout lengths remain well described, but short-term behaviors, such as ARS, are not consistently captured. Researchers often make logistical trade-offs for tag deployments between cost, battery power, tracking duration, recapture probability, and data resolution. Yet, sampling interval should not be sacrificed idly due to implications on the ability to record shorter-term behaviors. For instance, if fine-scale management schemes are to be derived from movement data, deployment durations may need to be sacrificed in favor of a higher sampling resolution.

RST’s value can be broadly extended toward habitat and distribution studies to better connect movement patterns with resource selection. To understand the behavioral mechanisms of animal space use, species distribution models and resource selection functions should be calibrated using behaviorally partitioned movement data [33]. Such partitioning can allow ecological questions to be addressed, such as elucidating environmental co-variates of resting and foraging areas, and how animals use wind, currents and topography during transit. RST can efficiently contribute to these efforts, allowing researchers to dedicate more time toward ecological models and interpretations. Although RST describes three discrete behavior groups (time & distance intensive positive points, time intensive negative points, and transit points where residuals equal zero), the residual values are continuous between -1 and 1, which offers more descriptive capacity of functional response curves derived by modeling studies. Furthermore, the confidence of behavior state assignment of each point by RST can be described by examining the mean and sd of residuals across variable *R*, enabling the identification of locations with simultaneously mixed behavior states (e.g., transit and ARS) or locations in transition between behavior states. As expected with hierarchical analyses, RST behavior groupings, as described by residuals, change with scale (Fig 7) and quantifying confidence of each point assignment as described here will help movement ecologists move away from identification of dichotomous behavior states and toward a more continuum approach to behavior description (e.g., [32]). Additionally, the normalized and continuous range of RST residuals allows for further examination based on range, clusters, percentage and intensity to compare patterns across individuals, populations, seasons, habitat, life-history groups and movement association with anthropogenic entities (e.g., fishing vessels, trash dumps, urban areas).

Unlike most other behavioral classification methods, RST’s functionality is based on classification of transit points (residuals = 0) as determined by the choice of *R*. These transit points then partition time & distance intensive positive residuals from time intensive negative residuals. Interestingly, while these positive and negative residuals identify groups of behaviorally similar points within a track, it is up to the user to interpret the meaning of this time & distance intensive and time intensive classification based on scale and ecological knowledge of the study species. For example, while time intensive points indicate where the animal spent more time and less distance within the analysis circle relative to other areas where distance traveled was larger, these negative residuals are interpreted as rest locations in our fine-scale albatross track example, but are more likely areas of concentrated feeding behavior in the larger scale blue whale track. Locations with positive residuals along both the albatross and blue whale tracks indicate where distance traveled was relatively larger at the scale of analysis and therefore describe more intensive searching behaviors, but at two different scales. Additionally, due to the great diversity of how animal movement patterns relate to behavior state, such as the unusual resting behavior of frigate birds (*Fregata minor*) while in flight [34], the RST user must interpret the meaning of residuals based on the scale of analysis and the study animal’s ecology.

As a new method, we promote the cross assessment of RST relative to other movement data behavior analyses, as these efforts frequently reveal the strengths and weaknesses of various approaches [14, 35]. To focus analyses and limit time investment, it is important to understand nuance in both the behavior of the tracked animal and the dataset to be analyzed prior to implementing hypothesis testing and computationally intensive analysis. It is here that the RST method can provide insight into the individuality of each track. Furthermore, we encourage other researchers to implement RST on movement data across taxa, scales and ecosystems to examine method performance and to conduct meta-analyses. With diverse datasets, if a desired scale of analysis is undefined, application of the track-specific dynamic scaling approach will allow description of scale consistency across the movement datasets and identification of outliers that require data exploration and possible correction. Once reliable RST behavior classifications are derived for each track, then comparisons are feasible due to normalized values of RD, RT and residuals. Additionally, complimentary biologging, such as immersion, accelerometer, and time-depth recorder data, can be used to further describe taxa specific behaviors and movements related to the residual results (e.g., [6]) or incorporated into the RST method. For example, RST could be extended from 2D to 3D by converting from a circle to a sphere-based analysis, complimentary to spherical first passage time [36].

### RST recommendations

The RST code is freely available (S3 Appendix) and we recommend the following initial settings: Implement dynamic scaling approach with a range of *R* based on prior knowledge of animal movement patterns and scale of sampling (how far is the animal likely to move between locations?); visually inspect the classification of the tracks; assess the consistency of choice of *R* across individual tracks; investigate tracks with outlier values for *R*; interpret states. Despite these recommendations, no one-setting fits all data, but RST analysis of movement data is fast, allowing users the freedom to iterate analyses to test and refine parameters; this flexibility allows the user to hone in on the behavioral profile of interest and appropriate spatio-temporal scales, thus focusing subsequent analyses [14].

## Conclusions

Animal tracking is revolutionizing our understanding of animal ecology in a myriad of ways including behavior, social systems, habitat use, and population connectivity. Yet, choosing and applying the appropriate analytical method can be challenging and cumbersome, making the simplest approach often the most desirable [11, 14, 37]. The RST method offers an intuitive, rapid, iterative and flexible approach to explore movement data, with limited *a priori* assumptions (except the assumption that the sampling interval of the data is low enough to capture meaningful movement behaviors), that can assist more sophisticated explanatory and predictive analyses [14]. As a stand-alone method, RST analysis provides the ability to standardize movement data exploration across taxa, ecosystems, and data-types, offering immense opportunities for meta-analyses and initial steps toward answering pressing ecological questions regarding animal movement drivers, response and scale.

## Supporting Information

S1 Appendix Probability of equal residual value resulting from different combinations of Residence Distance (RD) and Residence Time (RT). (DOCX)

S2 Appendix Temporal sub-sampling of gray-headed albatross GPS tracks using Residence in Space and Time (RST) method. (DOCX)

S3 Appendix Zip file containing R code, documentation and example dataset for running Residence in Space and Time (RST) method. (ZIP)
