# Supplementary material for: Classification of Animal Movement Behavior through Residence in Space and Time
Source: PLoS One. 2017 Jan 3;12(1):e0168513. doi: 10.1371/journal.pone.0168513 (PMC5207689; doi:10.1371/journal.pone.0168513)
Supplement: S1 Appendix — (DOCX) [file pone.0168513.s001.docx]

**Animal movement analysis through residence in space and time, Torres et al.**

S1 Appendix: Probability of equal residual value resulting from different combinations of Residence Distance (RD) and Residence Time (RT)

Here we examine the probability that the same residual value will result from different combinations of Residence Distance (RD) and Residence Time (RT) using the Residence in Space and Time (RST) approach:

Formula 1 *Residuals = ((RD) / (max. RD of the track)) - ((RT) / (max. RT of the track))*

The probability of the same resulting residual value will depend on the distributions of RD and RT. To illustrate this relationship, we show the distribution of normalized RD and RT values for the example grey-headed albatross GPS track (Bird 23059; Fig. 1) and all incubation albatross tracks (Fig. 2). Overlaid on these figures are lines corresponding to resulting residual values derived by Formula 1. The points under any given line have equal residual values. These plots demonstrate how often different pairs of RD and RT result in the same residual value, and their distribution.


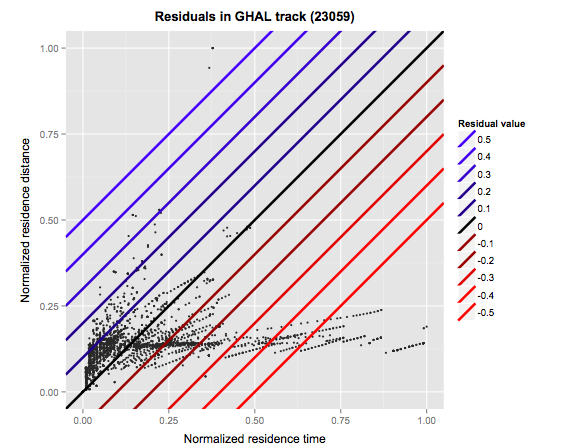


Figure 1. The distribution of normalized residence distance and normalized residence time values from grey-headed albatross GPS track 23059, with lines overlaid that correspond to resulting residual values derived by Formula 1. The lines are color-coded to correspond to the representative colors of positive (blue), negative (red), and zero (black) residual values.


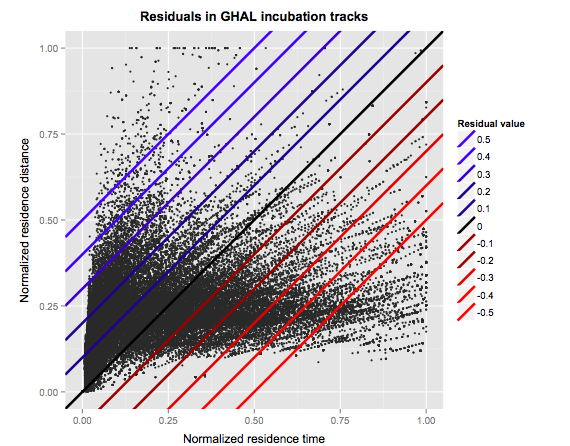


Figure 2. The distribution of normalized residence distance and normalized residence time values from all incubation grey-headed albatross GPS tracks (n=94,742 locations), with lines overlaid that correspond to resulting residual values derived by Formula 1. The lines are color-coded to correspond to the representative colors of positive (blue), negative (red), and zero (black) residual values.
