# Supplementary material for: Classification of Animal Movement Behavior through Residence in Space and Time
Source: PLoS One. 2017 Jan 3;12(1):e0168513. doi: 10.1371/journal.pone.0168513 (PMC5207689; doi:10.1371/journal.pone.0168513)

**Animal movement analysis through residence in space and time, Torres et al.**

S2 Appendix: Temporal sub-sampling of gray-headed albatross GPS tracks using Residence in Space and Time (RST) method.

Because the GPS loggers did not acquire a fix every 5 min (5.63 ± 0.59 min, n=24), tracks were linearly interpolated between short gaps greater than 8 minutes to aid in temporal subsampling. We chose this option to retain the original GPS data points. Regardless, intervals are not exact but approximate.

Table 1. Sample intervals ± SD and radii ± SD applied in the Residence in Space and Time (RST) method of all albatross tracks using the dynamic scaling approach for subsampled tracks.

| Sample Interval (min) | Radius (km) |
| --- | --- |
| 5.29 ± 0.43 | 2.40 ± 0.36 |
| 10.58 ± 0.85 | 4.47 ± 0.72 |
| 21.16 ± 1.71 | 8.43 ± 1.70 |
| 31.75 ± 2.56 | 13.23 ± 2.79 |
| 63.50 ± 5.13 | 23.04 ± 5.33 |
| 127.01 ± 10.26 | 41.52 ± 11.43 |
| 190.5 ± 15.39 | 60.43 ± 20.28 |

Figure 1. Comparison between one grey-headed albatross GPS track (Bird 23059) at (A) the 5 min interval resolution (lineally interpolated across short gaps, 4.96 ± 0.48 min) and subsamples (B) 10 min (9.91 ± 0.87 min), (C) 20 min (19.83 ± 1.49 min), (D) 30 min (29.75 ± 2.03), (E) 60 min (59.49 ± 3.49 min), (F) 120 min (118.99 ± 5.45), and (G) 180 min (178.48 ± 6.78).

A) 5 min


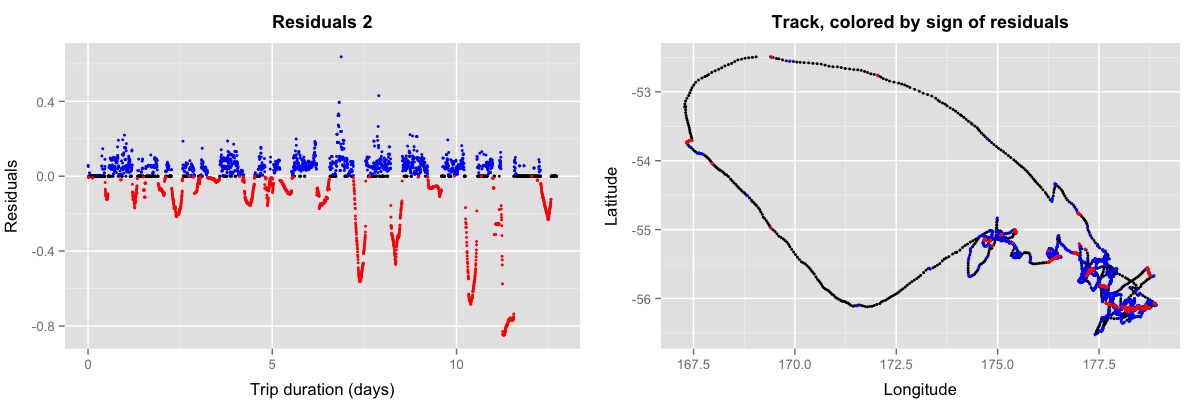


B) 10 min


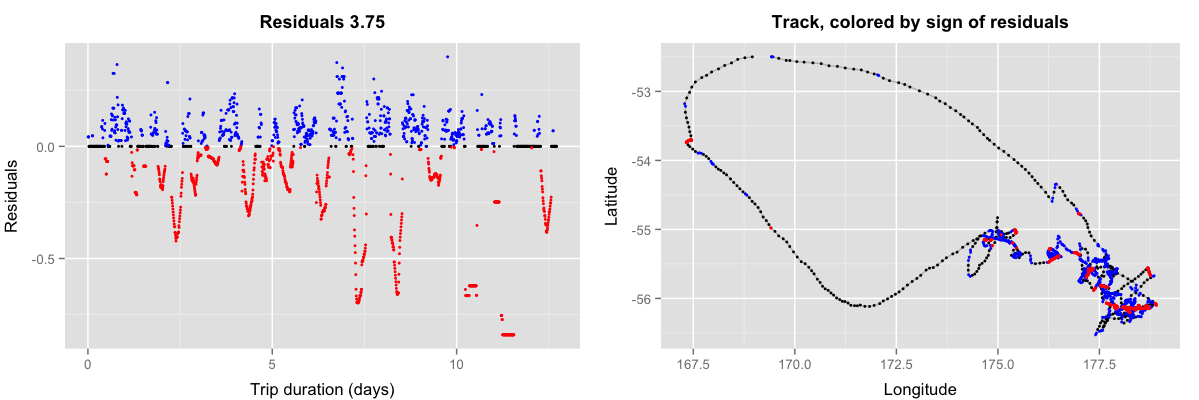


C) 20 min


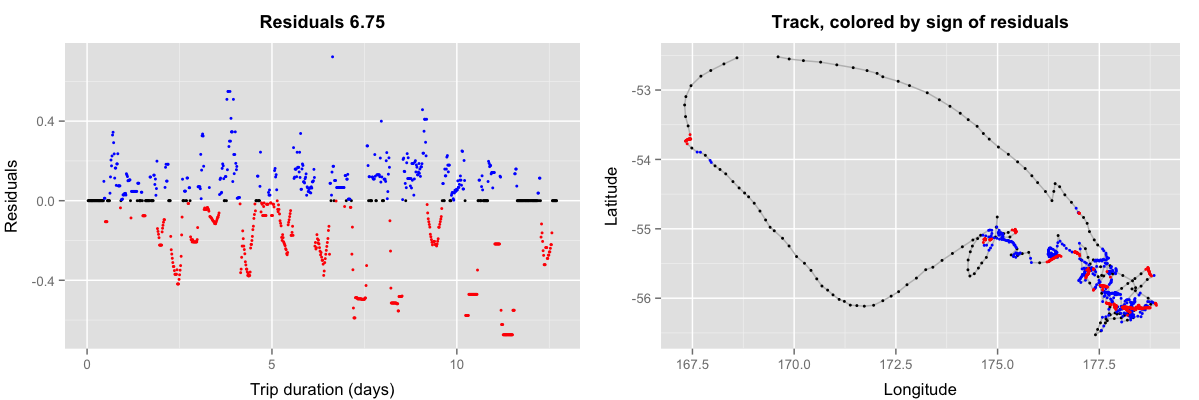


D) 30 min


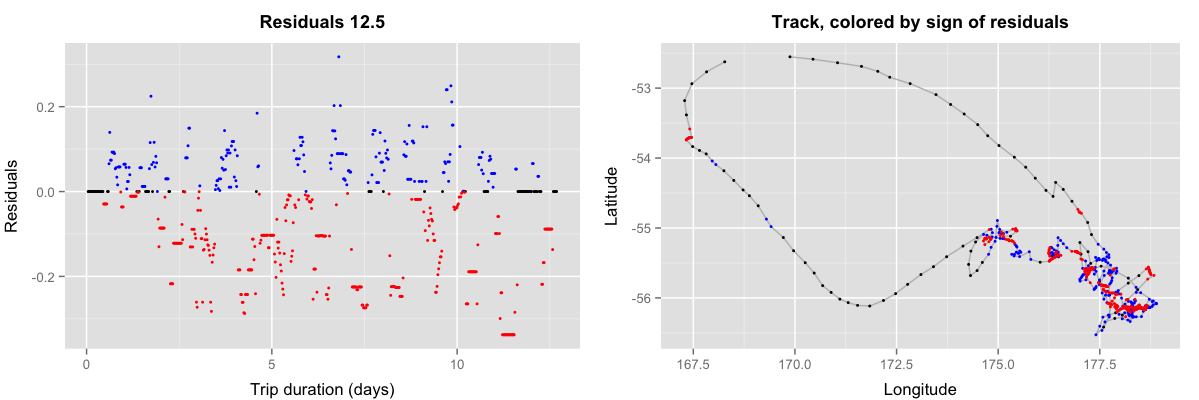


E) 60 min
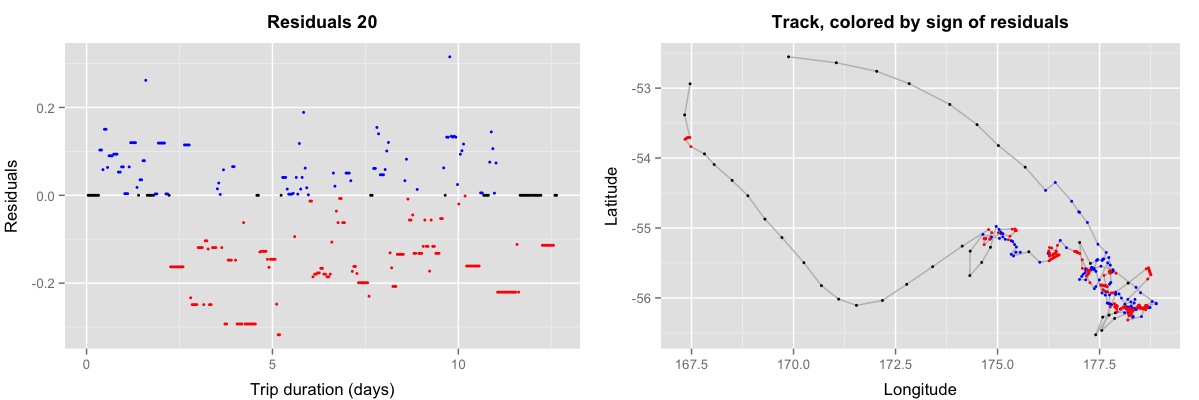


F) 120 min


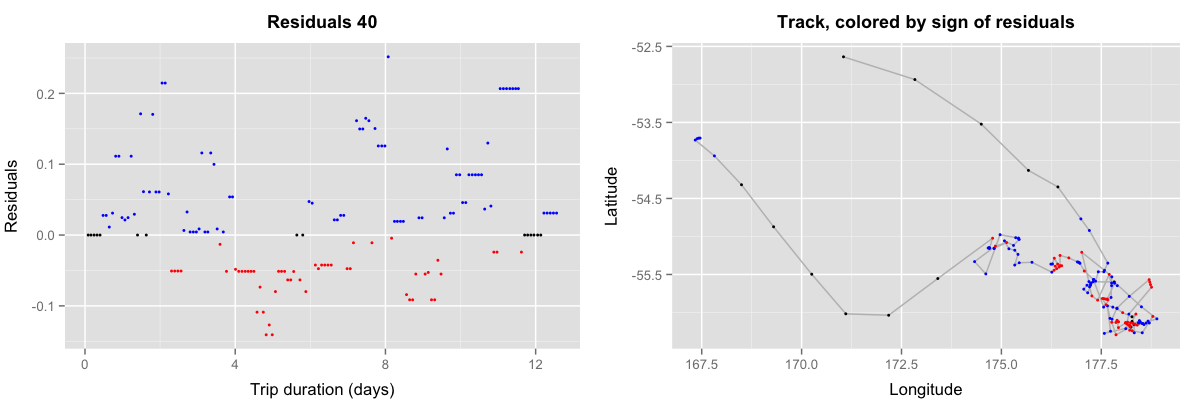


G) 180 min


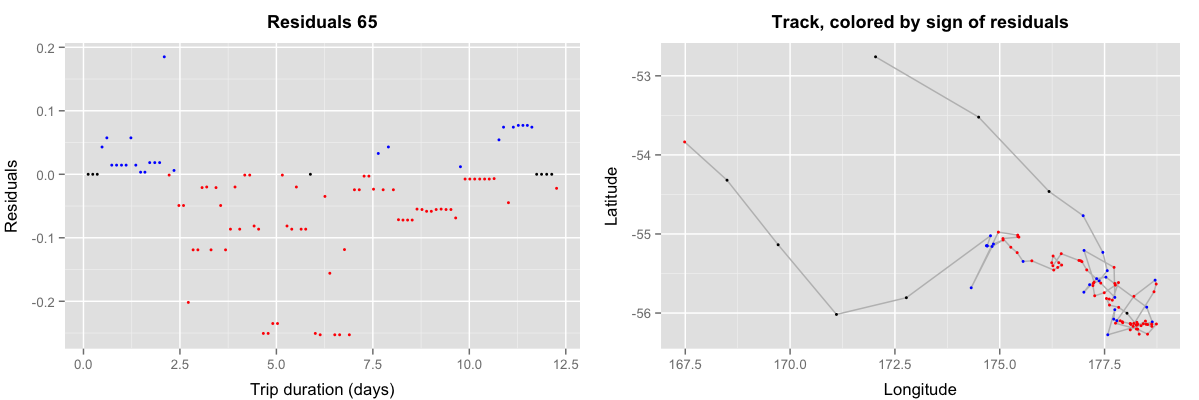


Figure 2. Original 5 min grey-headed albatross track (Bird 23059; lineally interpolated across short gaps) compared to three examples of simulated satellite telemetry data by stochastically sampling 1/3 of locations along the 60 min interval track (see Figure 1E).


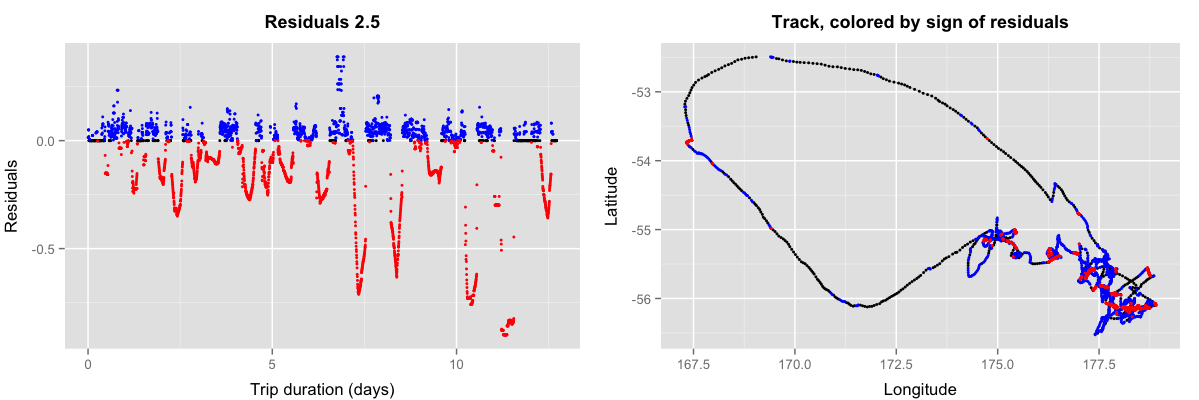


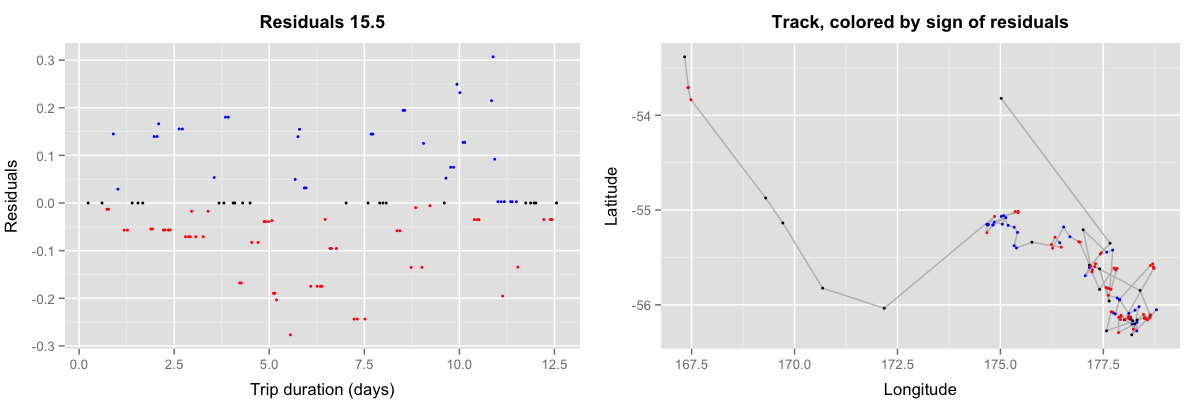

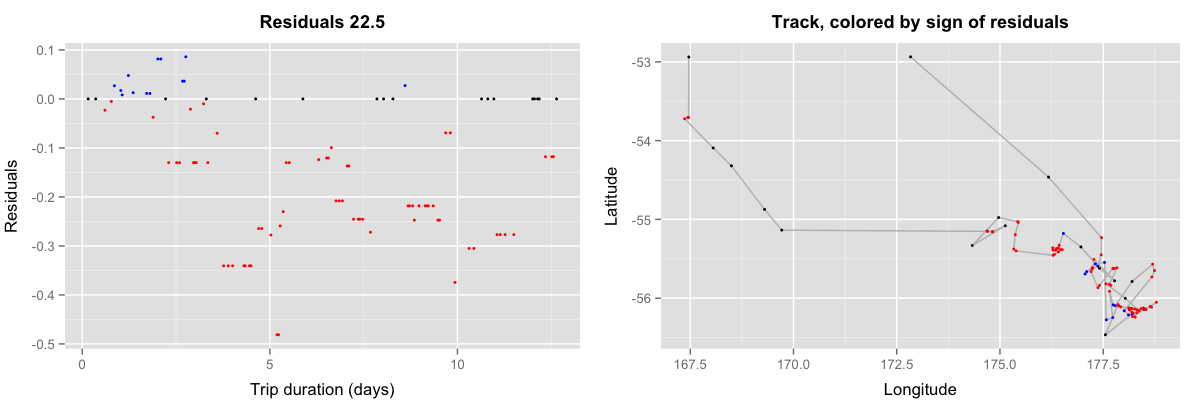

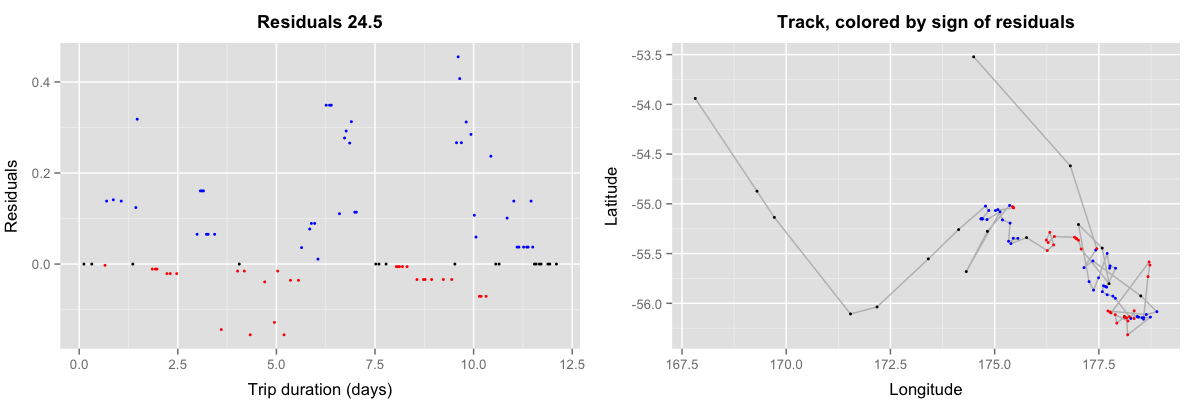

Supplement: S2 Appendix — (DOCX) [file pone.0168513.s002.docx]
