# Supplementary material for: Classification of Animal Movement Behavior through Residence in Space and Time
Source: PLoS One. 2017 Jan 3;12(1):e0168513. doi: 10.1371/journal.pone.0168513 (PMC5207689; doi:10.1371/journal.pone.0168513)
Supplement: S3 Appendix — (ZIP) [file pone.0168513.s003.zip › Supplement.docx]

**Animal movement analysis through residence in space and time, Torres et al.**

S3 Appendix: R code and example dataset for running Residence in Space and Time (RST) method

**File list**

GHAL_23059.csv GPS track of a grey-headed albatross (example movement dataset used in paper).

RST_documentation Documentation and description of R functions used in RST analysis method.

RST_functions_all R source code for all functions used in RST analysis method.

RST_general_tracks R code for the user to manipulate to input dataset, set parameters and run RST analysis.

RST_dynamic_tracks R code for the user to manipulate to input dataset, set parameters and run RST analysis using the dynamic scaling methods to choose radius scale.

RST_residenceRadii.so C code to implement R functions for RST analysis (for mac computers)

RST_residenceRadii.dll C code to implement R functions for RST analysis (for PC computers)

**Description**

In this supplement we provide code to conduct the Residence in Space and Time (RST) analysis method of animal movement data, and the example grey-headed albatross (GHAL) GPS track used in this paper (Bird 23059; GHAL_23059.csv). All analysis is completed in R (R Development Core Team 2013) and implemented in C. The following packages are utilized: ggplot2 (Wickham 2009), mapdata (Brownrigg 2015), and mapproj (McIlroy 2015).

Column definitions of GHAL_23059.csv

1. SortID: a unique identifier for each row of data
2. band: a unique identifier for each tracked animal (in this example there is only one albatross tracked, but the user may analyze a data file with tracks from multiple indiviudals).
3. Datetime_GMT: The date and time stamp of each location point. This may be in multiple formats, with subsequent format definition with strptime in the R code (RST_general_tracks.R)
4. lat: Latitude of each location (in decimal degrees)
5. lon: Longitude of each location (in decimal degrees)

To implement the RST code the user must:

- Have the packages ggplot2 and mapproj installed in R.
- Use the “RST_general_tracks.R” script to apply user defined radius (*R*) values or “RST_dynamic_tracks.R” to allow *R* to be selected for each track based on the radius where <5% of points are transit (residual = 0).

**Literature cited**:

Brownrigg, R. 2015. mapdata: Extra map databases. GPL-2.

McIlroy, D. 2015. mapproj: Map projectuins. Lucent Public License.

R Development Core Team. 2013. R: A language and environment for statistical computing. R Foundation for Statistical Computing, Vienna, Austria.

Wickham, H. 2009. ggplot2: elegant graphics for data analysis. Springer Science & Business Media.
