# Supplementary material for: Classification of Animal Movement Behavior through Residence in Space and Time
Source: PLoS One. 2017 Jan 3;12(1):e0168513. doi: 10.1371/journal.pone.0168513 (PMC5207689; doi:10.1371/journal.pone.0168513)
Supplement: S3 Appendix — (ZIP) [file pone.0168513.s003.zip › RST_documentation.docx]

**RESIDENCE IN SPACE AND TIME**

Nov 22, 2015

| residenceCalc *calculates the residence time and distance for a dataset* |
| --- |

**Description**

This method calculates the residence time and distance for a given dataset and scale.

**Usage**

residenceCalc (x, y, time, radius, threshold)

**Arguments**

| x, y | The x and y arrays give the x- and y- grid coordinates of the tracking points. These must be from coordinates over a grid, not over a sphere (not longitude and latitude). |
| --- | --- |
| time | The time array gives the points in time at which each measurement was taken. This must be a continuous quantity, such as the number of seconds, minutes, days, etc, that have passed since the start of data collection. The residence time calculations will be in these units. |
| radius | This array gives the radii of the circle taken around each point for finding the residence time and distance of a given point. When multiple radii are given, the analysis is repeated for each value.  This array must be in the same units as the x and y coordinates. |
| threshold | The threshold defines the maximum allowable deviation outside of a circle before re-entry such that the distance and time traveled will be included in the residence value for a given point. This array should either be a single number if a constant threshold is applied to all radii value, or should be the same length as the radius vector. |

**Value**

This method returns a data frame with the same number of rows as the number of entries in the x, y, or time arrays. There are five columns per each radius value. For example, if we use a radius of 10 km and a threshold of 2 km, the output will have the columns:

*scale_RT_rad_10_th_2*: the residence time of each point, calculated in the units of the time array

*scale_RD_rad_10_th_2*: the residence distance of each point, calculated in the units of the x and y arrays

*norm_RT_rad_10_th_2*: the normalized residence time, equal to the residence time divided by the maximum residence time value

*norm_RD_rad_10_th_2*: the normalized residence distance, equal to the residence distance divided by the maximum residence distance value

*res_rad_10_th_2*: the residence residuals, equal to normalized residence time subtracted from normalized residence distance

**Note**

The x- and y- arrays can be obtained by using a projection from spherical (longitude / latitude) coordinates to grid coordinates. The best projection to use may depend on the scale and location of analysis; see the *mapproj* package (Doug McIlroy) for more information.

The time array needed for this function can be obtained from a dates and times by converting the dates/times to a POSIX object and subtracting the time value of the first measurement. For further information, see the documentation of the *as.POSIX* and *strptime* functions.

For residence and residual calculations, NA are assigned to (1) locations at the beginning of a track until the animal moves beyond R from the initial point, and (2) to those locations at the end of a track that are all within R of the last radius built.

| haversineDist *calculates the distance between two lon/lat points* |
| --- |

**Description**

This method calculates the distance along the surface of the sphere between two (lon, lat) coordinates using the haversine formula.

**Usage**

haversineDist(lon1, lat1, lon2, lat2, rad = 6371)

**Arguments**

| lon1, lat1 | Coordinates of second point |
| --- | --- |
| lon2, lat2 | Coordinates of first point |
| rad | The Earth’s radius, in the units of choice. The default is set to kilometers, or *rad = 6371*. |

**Note**

The distance is calculated in kilometers by default, and alternate units can be specified by defining the Earth radius “rad” in those units. For example, to calculate distance in miles, set *rad = 3959*.

Longitude should range between -180 and 180 or between 0 and 360. Latitude should range between -90 and 90.

| plotTrackContour *plots the geographic track, color-coded by residence value* |
| --- |

**Description**

This method plots the tracks given in a dataset and creates a double plot, with the color-coding done by residence time (left) and residence distance (right).

**Usage**

plotTrackContour(tracks, radius, lwd = 1)

**Arguments**

| tracks | An output data set from the residence calculations, containing the time, x-position, y-position, residence time, residence distance, and residual values. |
| --- | --- |
| radius, thresh | The chosen radius and threshold pair for the residence values for a given plot. |
| lwd | Line width. |

**Note**

This method depends on correct naming of the columns in the “tracks” dataset: of the form “norm_RT_rad_10_th_5” (output from residenceCalc).

| plotTrackRes *plots a color-coded geographic track alongside the residuals* |
| --- |

**Description**

This method plots the tracks given in a dataset, color-coded by the sign of the residual, where a point’s residual is the difference between the normalized residence time and distance. The first plot shows the residual values against time and the second shows the geographic track.

**Usage**

plotTrackRes(tracks, radius, time_units, ps = 5)

**Arguments**

| tracks | An output data set from the residence calculations, containing the time, x-position, y-position, residence time, residence distance, and residual values. |
| --- | --- |
| radius | The chosen radius of residence calculations for a given plot. |
| time_units | Time units corresponding to the time calculations from the residence calculations (can be "secs", "mins", "hours", "days", "weeks"). |
| ps | Pointsize (default set to 5). |

**Note**

This method depends on correct naming of the columns in the “tracks” dataset: of the form “norm_RT_rad_10_th_5”.

In these plots blue points indicate positive residual values (distance intensive points), red points indicate negative residual values (time intensive points), and black points indicate residuals with a value of 0 (transit points).

| plotMultiScale *plots the proportion of each Residual type (Positive, Negative, and Zeros) for a user selected range of radius values with one plot for each track allowing side-by-side comparisons. Output is needed to run function ‘chooseDynScale’.* |
| --- |

**Description**

This method plots the proportion of each residual group (Positive = blue, Negative = red, and Zeros = blue) for a user selected range of radius values. This function is intended to plot multiple individuals on the same gridded plot. See function ‘plotMultiScale1’ if you are working with one track. The package ‘dplyr’ is a dependency.

**Usage**

plotMultiscale(tracks, radius)

**Arguments**

| tracks | An output data set from the residence calculations, containing the time, x-position, y-position, residence time, residence distance, and residual values. |
| --- | --- |
| radius | A numeric vector (in numerical order) of radius values selected to determine data-determined choice of radius scale. |

**Note**

This method depends on correct naming of the columns in the “tracks” dataset for the residual columns to contain the word ‘res’. Output from either ‘plotMultiScale’ or ‘plotMultiScale1’ is needed for function ‘chooseDynScale’.

| plotMultiScale1 *same as function ‘plotMultiScale’ but intended for one track (output resembles Fig 2c, Torres et al.).* *Plots the percentage of each Residual type (Positive, Negative, and Zeros) for a user-selected range of radius values as multiplot. Output can be used for function ‘chooseDynScale’.* |
| --- |

**Description**

This method plots the proportion of each residual group (Positive = blue, Negative = red, and Zeros = blue) for a user selected range of radius values. This function is intended to plot one track.

**Usage**

plotMultiscale1(track, radius, xmax, Resti=-10)

**Arguments**

| track | An output data set from the residence calculations, containing the time, x-position, y-position, residence time, residence distance, and residual values. |
| --- | --- |
| radius | A numeric vector (in numerical order) of radius values selected to determine data-determined choice of radius scale. |
| xmax | Maximum value for the x-axis on a scale plot. Good for zooming in. |
| Resti | Estimated preferred radius value. If none is known default is set to -10 so this part won’t plot. |

**Note**

This method depends on correct naming of the columns in the “tracks” dataset for the residual columns to contain the word ‘res’. Output from either ‘plotMultiScale’ or ‘plotMultiScale1’ is needed for function ‘chooseDynScale’.

| chooseDynScale *This method selects the radius value where the proportion of transit points first goes below 0.05.* |
| --- |

**Description**

This method selects the radius value where the proportion of transit points first goes below 0.05.

**Usage**

chooseDynScale(scales, radius)

**Arguments**

| scales | An output data set from either plotMultiScale1 (one track) or plotMultiScale. |
| --- | --- |
| radius | A numeric vector (in numerical order) of radius values used to run plotMultiScale1 or plotMultiScale. The range of radius values must be chosen so that they extend across the point where the proportion of transit points drops below 0.05. Additionally, it is helpful to increase the resolution of radii increments near where the proportion of transit points falls below 0.05. |

**Note**

This method depends on the output from plotMultiScale1 or plotMultiScale. Animal or track ids must be numeric.

| plotTrackResDyn *plots a color-coded geographic track alongside the residuals of a track with a dynamically chosen radius.* |
| --- |

**Description**

This method plots the tracks given in a dataset, color-coded by the sign of the residual, where a point’s residual is the difference between the normalized residence time and distance. The first plot shows the residual values against time and the second shows the geographic track. The radius value is selected from the output of ‘chooseDynScale’.

**Usage**

plotTrackRes(id, all_tracksD, dynscale, time_units, ps = 1)

**Arguments**

| id | Numeric track id. |
| --- | --- |
| all_tracksD | An output data set from the residence calculations, containing the time, x-position, y-position, residence time, residence distance, and residual values. Contains all tracks and is an output from dynamically selected radius scale. |
| dynscale | The output from ‘ChooseDynScale’ that contains the chosen radius of residence calculations for all_tracksD. |
| time_units | Time units corresponding to the time calculations from the residence calculations (can be "secs", "mins", "hours", "days", "weeks"). |
| ps | Pointsize (default set to 1). |

**Note**

This method depends on correct order of the columns in the “all_tracksD” dataset: where the residuals are in the 14^th^ column.

In these plots blue points indicate positive residual values (distance intensive points), red points indicate negative residual values (time intensive points), and black points indicate residuals with a value of 0 (transit points).
